# Platypnea‐orthodeoxia syndrome in a patient with ongoing COVID‐19

**DOI:** 10.1002/rcr2.1009

**Published:** 2022-07-19

**Authors:** Maki Asami‐Noyama, Misa Harada, Yukari Hisamoto, Taiga Kobayashi, Keiji Oishi, Nobutaka Edakuni, Tsunahiko Hirano, Tomoyuki Kakugawa, Kazuto Matsunaga

**Affiliations:** ^1^ Department of Respiratory Medicine and Infectious Disease Yamaguchi University School of Medicine Graduate School of Medicine Ube Japan; ^2^ Department of Radiology Yamaguchi University School of Medicine Graduate School of Medicine Ube Japan; ^3^ Department of Pulmonary and Gerontology Yamaguchi University School of Medicine Graduate School of Medicine Ube Japan

**Keywords:** COVID‐19, perfusion scintigraphy, platypnea‐orthodeoxia syndrome, V/Q mismatch

## Abstract

SARS‐CoV‐2 infection of the vascular endothelium causes excessive vasodilation. It is important in the rehabilitation of patients with COVID‐19 to recognize that increased blood flow in lung lesions at the base of the lung due to vasodilation may cause V/Q mismatch and result in platypnea‐orthodeoxia syndrome.

## Graphical abstract

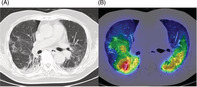

## CLINICAL IMAGE

**FIGURE 1:**
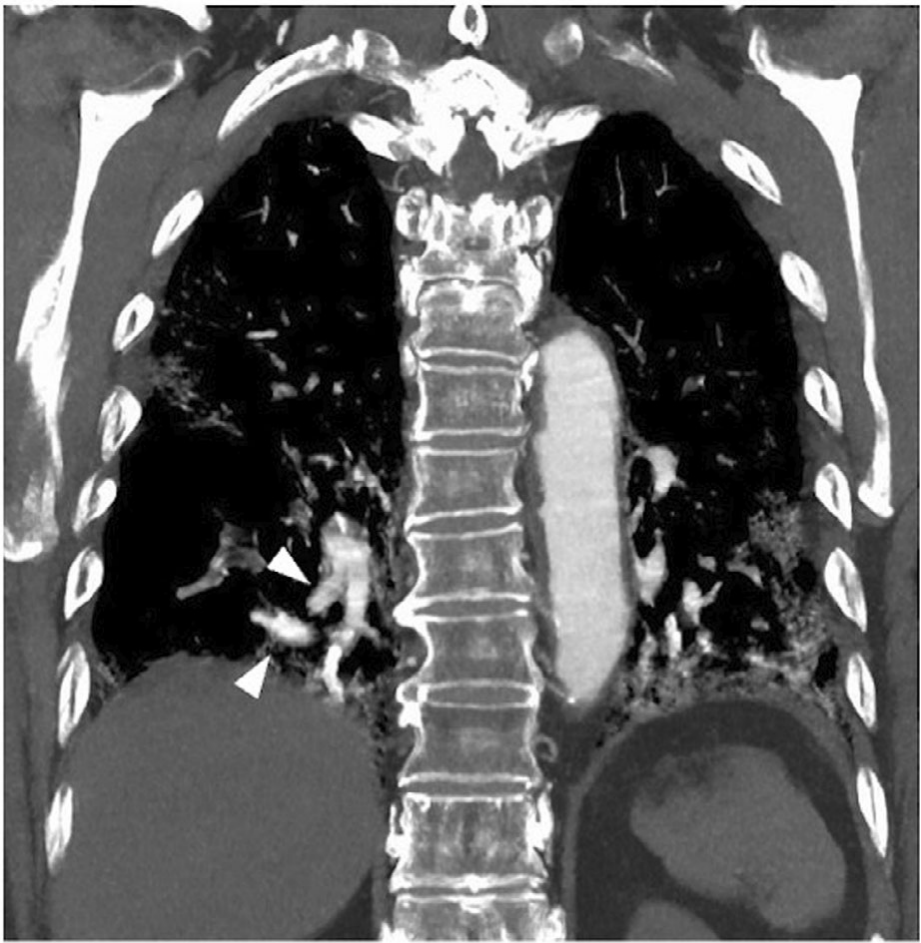
A contrast CT scan revealed an obvious vascular enlargement toward the lesions in the lower lobe bilaterally (arrowhead). CT, computed tomography

**FIGURE 2:**
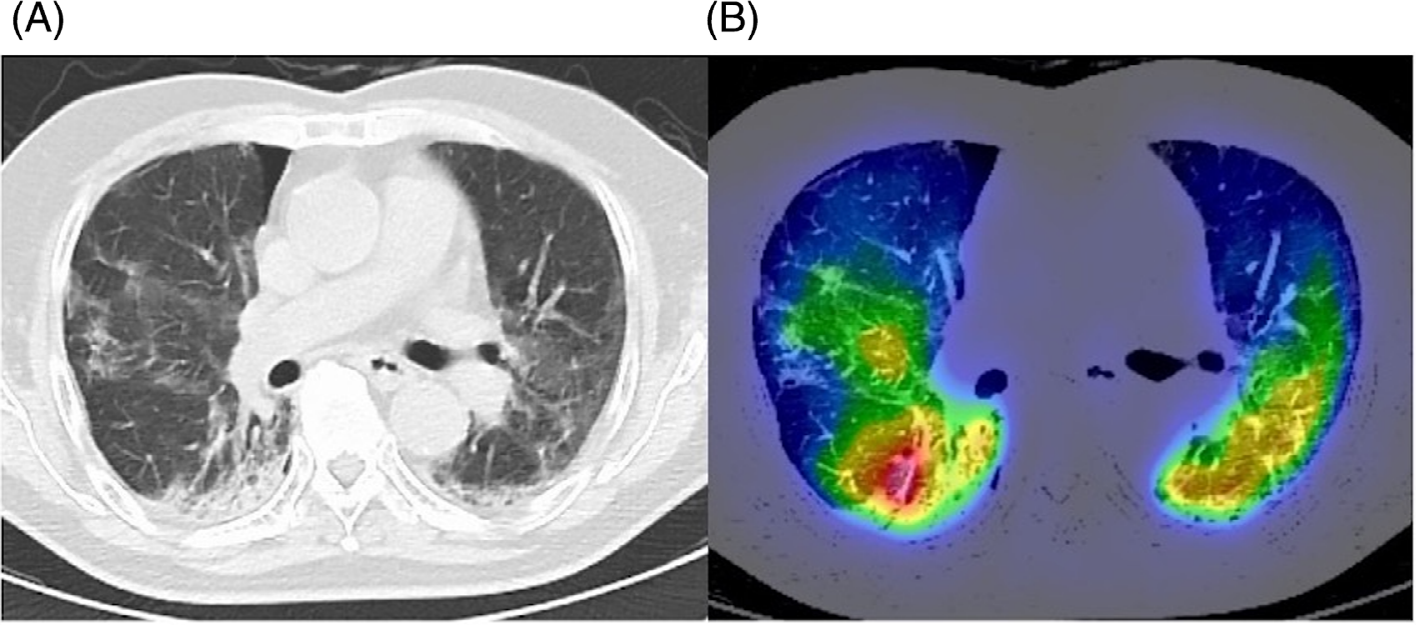
(A, B) A ventilation/perfusion single‐photon emission computed tomography combined with computed tomography (V/Q SPECT/CT) showed perfusion uptake within and around the fibrotic lesions in the lower lobe bilaterally. CT, computed tomography

A 83‐year‐old male with ongoing COVID‐19 was diagnosed with platypnea‐orthodeoxia syndrome (POS) 1 month after infection. He had type 2 diabetes mellitus and angina pectoris. He was an ex‐smoker (28‐pack years). His initial chest computed tomography (CT) scan showed bilateral ground glass opacities. Dexamethasone and baricitinib were administered on day 6, but oxygenation worsened, and he was transported to our hospital on day 11. He required oxygen therapy with a high‐flow nasal cannula (FiO_2_: 75%, flow: 40 L/min). Steroid pulse therapy was given on day 18–20, and oxygenation gradually improved. Blood tests showed a decreased inflammatory response (WBC 8130/μl, CRP 0.94 mg/dl). On day 27, he was transferred to the general ward for rehabilitation. Upon careful observation, we noticed that his oxygenation turned out to be significantly worse in the sitting position (PaO_2_ 64.8 mmHg) compared with the supine position (PaO_2_ 101.4 mmHg), with an oxygen flow rate of 3 L/min via an oxymizer cannula. Bubble‐contrast echocardiography revealed neither intracardiac nor intrapulmonary shunt. A contrast CT scan revealed a vascular enlargement toward the lesions (Figure 1). Vascular enlargement within the lesions is often seen in SARS‐CoV2 pneumonia.^1^ A ventilation/perfusion single‐photon emission computed tomography combined with computed tomography (V/Q SPECT/CT) showed perfusion uptake within and around the fibrotic lesions (Figure 2A,B). It was speculated that the sitting position would increase blood flow to the fibrotic‐like lesions at the lower zones, where ventilation is reduced, and make the V/Q mismatch more prominent. V/Q mismatch within the lesions leading to POS may occur in some cases of ongoing COVID‐19 pneumonia. Two weeks later, the patient was transferred with gradual improvement in oxygenation, but POS remained.

## AUTHOR CONTRIBUTION

Maki Asami‐Noyama, Misa Harada and Yukari Hisamoto were engaged in medical treatment as attending physicians. Taiga Kobayashi pointed out features of the imaging findings. All authors reviewed the manuscript draft and revised it critically on intellectual content. All authors approved the final version of the manuscript to be published.

## CONFLICT OF INTEREST

None declared.

## ETHICS STATEMENT

The authors confirm that appropriate written informed consent was obtained for the publication of this manuscript and accompanying images.

## Data Availability

No data are available.
